# High-resolution imaging techniques to interrogate virus-host interactions

**DOI:** 10.1128/msphere.00816-25

**Published:** 2026-02-19

**Authors:** Isabel Amaya, Chelsey C. Spriggs

**Affiliations:** 1 Department of Cell and Developmental Biology, University of Michigan Medical School 12266 , Ann Arbor, Michigan, USA; 2 Department of Microbiology and Immunology, University of Michigan Medical School 12266 , Ann Arbor, Michigan, USA; 3 Life Sciences Institute, University of Michigan 123743, Ann Arbor, Michigan, USA; Virginia-Maryland College of Veterinary Medicine, Blacksburg, Virginia, USA

**Keywords:** virus-host interactions, high-resolution microscopy, virology research

## Abstract

Viruses are obligate parasites that rely extensively on host cellular machinery to complete their life cycles. Therefore, examining the fundamental nature behind how viruses interact with their hosts can teach us more about the pathogenesis of these microbes while also contributing to our overall understanding of essential cell biological processes. In a 2021 mSphere of Influence article, the use of live-cell imaging to uncover a unique mechanism of viral assembly was discussed. In this Full Circle review, we highlight the high-resolution imaging techniques currently revolutionizing virology research and discuss how they can be utilized to advance our ability to identify and interrogate novel virus-host interactions.

## INTRODUCTION

For decades, microscopy has been considered an essential tool in virology research. In fact, its various applications have been used to dissect the underlying principles behind virtually every step of viral infection, including receptor binding, internalization, trafficking, replication, assembly, and egress. While biochemical and molecular biological techniques certainly add to our understanding of virus-host interactions, the ability to visualize what is happening inside an infected cell is invaluable for clarifying key aspects of the viral life cycle and for generating new hypotheses to drive research forward. Moreover, observing the unique cellular changes that occur during viral infection may also reveal novel principles of cell biology.

In a mSphere of Influence article (1), two papers by Procter et al. that perfectly demonstrate the benefits of using advanced microscopy to investigate virus-host interactions were discussed. In the first study, the authors sought to understand how the human cytomegalovirus (HCMV) forms a unique, yet poorly understood, virion assembly compartment (AC) in the cytoplasm of infected cells (2). By using live-cell imaging to study AC formation, they made several discoveries that transformed our understanding of both virus and host cell biology. First, they observed that the AC acts as a Golgi-derived microtubule-organizing center that manipulates discrete functions of microtubule plus-end binding proteins for its function. Unexpectedly, they also found that the nuclei of infected cells exhibit a persistent rotation that is required for the maturation of newly assembled virions. Their follow-up study dove deeper into this virus-induced phenomenon and revealed new insight into the role of acetylated microtubules, nuclear membrane complexes, and the nucleoskeleton in HCMV infection while also adding to our understanding of normal cellular nuclear positioning and polarity (3). Importantly, these studies reaffirmed the power of observation to scientific advancement and the value of using viruses to understand cell biology.

Live-cell imaging is still an important tool for studying the dynamic nature of viral infections, especially in the context of transient interactions between the virus and the cell. However, as technology has continued to advance, several additional methodologies have also emerged, each with their own unique applications. This review will highlight just some of the advanced imaging techniques that are now available for virology research. Here, we give a brief overview of each technique while discussing their advantages and limitations in examining virus-host interactions at high resolution (Table 1).

**TABLE 1:** Summary of the benefits and limitations of each of the discussed techniques^*a*^

Technique name	Resolution	Benefits	Limitations	Reference protocol
Expansion microscopy	70 nm (4.5 in linear dimension)	Standard reagent, user-friendly sample preparation, can be combined with other super-resolution techniques	Signal loss, sample fragility, and non-uniform expansion	Expansion microscopy: principles and uses in biological research (4)
SIM	100 nm	Generates high-resolution videos of living cells, compatible with conventional fluorescent probes and protocols	Requires post-imaging processing; artifacts can be difficult to detect	Strategic and practical guidelines for successful structured illumination microscopy (5)
STED	20 nm	Reduces colocalization artifacts, compatible with live-cell imaging	Requires various laser adaptations, can cause photobleaching/phototoxicity	STED super-resolved microscopy (6)
dSTORM	20 nm in the *X* and *Y*, 50 nm in the *Z*	Use of commercially available fluorescent probes, provides quantitative data	Time-consuming data acquisition and antibody artifacts	Direct stochastic optical reconstruction microscopy with standard fluorescent probes (7)
FIB-SEM	<10 nm	Minimal image processing and high spatial resolution	Destructive to samples and slow imaging speed	Enhanced FIB-SEM systems for large-volume 3D imaging (8)
Cryo-EM	3–4 Å	No crystallization is required, captures states that are rare or intermediate	High cost of equipment and challenging sample preparation	Cryo-EM: a unique tool for the visualization of macromolecular complexity (9)
Cryo-ET	3–4 nm	Preserves samples in a close-to-native state, high-resolution 3D imaging	Complex sample preparation, low contrast images, and artifacts	Cryo-electron tomography: a long journey to the inner space of cells (10)
CLEM	2–6 nm with superimposed localization with a protein of interest	Labels viral/cellular factors to be identified by EM, can visualize specific cellular compartments or proteins	Difficulties with aligning images from various microscopes and sample preparation/preservation	Correlated light and electron microscopy: ultrastructure lights up! (11)

^*a*^ 3D, three-dimensional; CLEM, correlative light and electron microscopy; cryo-EM, cryogenic electron microscopy; cryo-ET, cryogenic electron tomography; dSTORM, direct stochastic optical reconstruction microscopy; FIB-SEM, ocused ion beam scanning electron microscopy; SIM, structured illumination microscopy; STED, simulated emission depletion.

## EXPANSION MICROSCOPY

Expansion microscopy (ExM) involves the physical magnification of preserved biological specimens to allow for the high-resolution imaging of these samples using conventional microscopes. Initially described in 2015, this technique uses a swellable polyelectrolyte hydrogel to isotropically expand cells and tissues by several magnitudes while still preserving accurate biological information (12). Although there are now many variations of this technique, the typical ExM protocol requires optimization of the following steps: labeling, gelation, digestion, expansion, and imaging (13). First, molecular handles or labels are covalently attached to molecules of interest to allow them to bind to the hydrogel. Second, the sample is immersed in a sodium acrylate monomer solution along with acrylamide and the crosslinker N-N′-methylenebisacrylamide. This forms the densely crosslinked polyelectrolyte hydrogel that couples the molecules to the polymer mesh. Next, the sample is homogenized by chemical denaturation, which can be done by heat, enzyme digestion, or treatment with various detergents. Finally, the sample is submerged in water, which triggers swelling through osmotic force that expands the specimen by up to ~100× in volume or ~4.5× in linear dimension. At this stage, further labeling can be performed to identify specific components (e.g., fluorescent antibodies and amplification chemistry), or iterative expansion can be rendered to gain even higher resolution. Because the final sample is ~99% water, the increased transparency and decrowding of densely packed structures typically allow for the high-resolution imaging of cells and even thicker tissues (4).

ExM has recently emerged as a valuable tool for investigating the spatiotemporal interactions occurring during viral infection. Given the resolution barrier in studying viral-host interactions, this technique allows for clearer visualization of viral proteins that may not be as easily detectable with traditional immunofluorescence protocols. Researchers have already utilized ExM to answer important questions about human immunodeficiency virus (HIV) infection. HIV is an RNA virus that infects human immune cells and can lead to acquired immunodeficiency syndrome. Therapeutics against HIV-1 rely on our continued understanding of the various cellular machineries that are exploited during viral infection, emphasizing the need to further investigate the mechanisms behind these interactions (14). Thus far, ExM has led to new insights into key steps of HIV-1 infection, including nuclear events and viral assembly.

For example, Petrich et al. used ExM to identify viral replication centers (VRCs) in the host cell nucleus (15). The integration of reverse-transcribed double-stranded DNA into the host genome is a defining characteristic of retroviral infection. Previous literature reported that incoming capsids/cores are directed to nuclear speckles where they are uncoated to release the reverse-transcribed viral DNA (16); however, the molecular targeting to and capsid uncoating within this nuclear subdomain remained poorly understood. Using ExM, the authors found that distinct HIV-1 capsid populations can be differentially visualized within host cellular nuclei (15). Both punctate structures that were presumed to represent single viral cores, as well as more diffuse capsid signals that are indicative of disassembling cores, could both be observed. These signals colocalized with different cellular components, such as the nuclear speckle marker SRRM2 and the nuclear protein cleavage and polyadenylation-specific factor 6 (CPSF6), suggesting that ExM could be used to distinguish between stages of HIV-1 replication. It also demonstrated that ExM can help resolve specific virus-host interactions that are otherwise masked by traditional immunostaining, such as the HIV-1 capsid interaction with CPSF6 (17). Importantly, this paper confirmed that both virus-host interactions and essential cellular structures are preserved during ExM imaging of virus infection.

Another step in the HIV-1 life cycle that has been studied using this technique is the assembly and release of newly replicated HIV-1 particles at the plasma membrane. While researchers used total internal reflection fluorescence microscopy to show that Gag, a viral structural polyprotein, accumulates and multimerizes at the plasma membrane, creating spherical particles that form prior to viral assembly and spread, it also highlighted a major gap in the mechanism of how viral and cellular proteins cluster at this site (18). This incorporation of the host cellular factors into the viral envelope is essential for the proper assembly of infectious viral particles and plays a role in regulating viral spread. Therefore, in a follow-up study, the authors turned to ExM to clarify this process. Using this technique, they showed that a previously identified phospholipid, phosphatidylinositol 4,5-bisphosphate, accumulates at the host cell surface near Gag and plays a direct role in recruiting and incorporating cellular transmembrane proteins that regulate virus spread (e.g., CD43, PSGL-1, and CD44) into the viral envelope (19).

Overall, the findings from these two studies highlight the benefit of using ExM to obtain higher-resolution images of essential viral-host interactions occurring during various steps of infection. Among the techniques highlighted in this review, ExM is unique in that it allows researchers to obtain “super-resolution” images using more commonly available microscopes, making it a more affordable and therefore accessible tool for virology researchers around the world.

## STRUCTURED ILLUMINATION MICROSCOPY

Since its initial innovation over 25 years ago (20), structured illumination microscopy (SIM) has become a widespread imaging tool in the biological sciences. This technology, which uses a series of structured sinusoidal patterns to illuminate an unknown biological sample, only requires a wide-field fluorescence microscope and is especially useful for obtaining super-resolution images of living cells (21). Here, samples are illuminated with spatially structured excitation light in the form of superimposed patterns (e.g., Moiré fringes). These periodic interference patterns, which are close to the optical diffraction limit, generate high-resolution imaging data that later can be reconstructed using available algorithms (22). Ultimately, the processing of multiple excitation readouts collected at various angles provides sample images with twice the normal optical resolution (~100 nm). Furthermore, SIM can be used to gather both two-dimensional (2D) and three-dimensional (3D) images, depending on the number of laser beams used to create the illumination pattern (either two or three beams, respectively). Now available in many imaging core facilities, SIM is another more accessible technique that can be used for investigating multiple stages of the viral life cycle, including genome replication.

Similar to HIV-1, polyomaviruses (PyVs) replicate in VRCs within the host cell nucleus (23). Laser scanning confocal microscopy (LSCM) shows that, in addition to viral proteins, host replication and repair factors are also recruited to these dynamic subnuclear domains to aid in the resolution of newly replicated circular viral genomes (23, 24); however, spatial resolution limits hinder further analysis of the organization and composition of these VRCs. Therefore, Peters and Garcea took advantage of 3D-SIM to examine murine PyV (MuPyV) VRCs in greater detail (25). In comparing images acquired using either LSCM or 3D-SIM, the latter revealed that MuPyV induces spatially distinct subdomains within its VRCs that are marked by the presence of either a bright signal (foci) for the viral large T antigen (LT) or the cellular RPA32 protein. Pulse-chase labeling with 5-ethynyl-2′-deoxyuridine revealed that the viral DNA signal initially associates with LT within the VRCs but relocalizes to RPA32 foci within the first hour after viral DNA synthesis. Furthermore, the VRC-associated RPA32 was hyperphosphorylated, indicating that it may correspond with the DNA damage response signaling that assists with downstream viral DNA processing (26). Overall, the authors’ use of 3D-SIM highlights how this technology can be applied to resolve the complexities in the function of these and other virus-host interactions during infection.

## STIMULATED EMISSION DEPLETION MICROSCOPY

Simulated emission depletion (STED) microscopy is a super-resolution fluorescence microscopy technique that has been used across various life sciences disciplines, including virology. This technique provides improved resolution over traditional confocal microscopy by utilizing a donut-shaped depletion laser that creates a smaller and more distinct field of view. Fluorophores outside of this excitation center remain in a darkened state that is unable to emit fluorescence (27). The key to STED microscopy is the two laser beams: an excitation laser beam and the donut-shaped depletion beam with which it is overlaid. The use of these two lasers allows researchers to narrow in on smaller imaging volumes by selecting for the region that is exclusively excited within the donut field of view. Similar to SIM, STED microscopy can generate both 2D and 3D images and is compatible with live-cell imaging (28). However, STED does not require further computational processing, making it one of the more user-friendly high-resolution imaging techniques for virology researchers to operate.

In fact, Baharom et al. took advantage of the improved resolution provided by this technique to visualize the early trafficking events of influenza A virus (IAV) (29). IAV entry requires that it transports through the endosomal network where the acidic environment triggers conformational changes in the viral glycoprotein hemagglutinin (HA) that lead to fusion of the viral and cellular membranes and, subsequently, capsid release into the cytosol (30). Dendritic cells (DCs) are antigen-presenting cells that are essential for immune surveillance against viral infection; however, they are also susceptible to IAV infection (31). Here, the authors sought to investigate whether IAV interacts with the endocytic machinery differently in DCs compared to the respiratory epithelial cells that it usually targets. For this, they implemented a three-color STED microscopy strategy to visualize IAV nucleoprotein with the early endosome marker, EEA1, and late endosome/lysosome marker, LAMP1. They found that IAV binds to the surface of DCs and is internalized within 5 minutes post-infection. After this initial entry step, they then tracked the virus through the early and late endosomal compartments until it reached the nucleus and showed that it follows a similar infection timeline to that seen in epithelial cells. Interestingly, the first figure of this study provides compelling data for how applying deconvolution algorithms to STED images removes colocalization artifacts that might be observed when using traditional confocal microscopy.

Another group used STED microscopy to investigate the complex mechanisms of arenavirus transmission. Lymphocytic choriomeningitis virus (LCMV) is the prototypic species of the *Mammarenavirus* genus, which includes several other highly pathogenic viruses (risk group 4) such as the Lassa and Junín viruses that cause severe hemorrhagic fevers in humans (32). Therefore, Byford et al. used LCMV as a model for understanding the cell-to-cell spread of these more pathogenic species, given their structural and functional similarities (33). In this study, the authors showed that LCMV can bypass traditional extracellular routes of infection (i.e., egress and subsequent entry into a new cell) by trafficking through tunneling nanotube (TNT) connections (33). STED microscopy was integral to this discovery in that it allowed them to directly visualize the viral GP-1 structural component of LCMV within the F-actin containing TNT filaments that stretch between neighboring cells. They further confirmed that viral RNA containing ribonucleoprotein complexes was also capable of moving through these TNTs, which may serve as a fundamental mechanism for the widespread dissemination of arenaviruses within their hosts. In all, these two studies demonstrate how this more accessible super-resolution imaging technique can be implemented to answer pressing research questions into how viruses interact with their host cells.

## DIRECT STOCHASTIC OPTICAL RECONSTRUCTION MICROSCOPY

Direct stochastic optical reconstruction microscopy (dSTORM) is another super-resolution microscopy technique that uses conventional organic fluorophores to image single molecules at an optical resolution of ~20 nm (7, 34). The use of photoswitchable fluorophores (e.g., carbocyanines, rhodamines, and oxazines) in the presence of reducing agents allows for their stochastic activation and detection over time. This signal, which is recorded initially as an image stack, depicts a fluorescence emission pattern that can be approximated with a Gaussian function based on the locations of the fluorophores across space and time (35). As a result, an artificial image can be reconstructed from a large number of single-molecule coordinates to provide a super-resolution image of the subject of interest.

So far, dSTORM has shown promise in visualizing viral structures, such as the arrangement of viral capsid proteins and interactions with host cellular machinery during early infection. One example comes from the study of how viruses interact with microtubules to traverse the host cell cytoplasm. Lyssaviruses, including the rabies virus (RABV), are RNA viruses that attack the nervous system and cause disease in animals and humans. RABV encodes a microtubule-associated protein called P3 that was previously shown to interact with microtubules and disrupt an antiviral immune response (36). However, the relationship between P3 and microtubules in the context of viral pathogenesis had not been directly examined. Toward this, Brice et al. used dSTORM to visualize the structural changes to single microtubule filaments during viral infection. They found that when comparing pathogenic and non-pathogenic RABVs, the non-pathogenic virus has impaired microtubule binding and IFN/STAT1 antagonism (37). Using dSTORM, they were able to visualize structural changes to microtubule architecture, which were specific to infection by the pathogenic RABV. Overall, the authors found that the rearrangement of the host cytoskeleton by the pathogenic RABV P3 is required for its interferon-antagonist function and is a key mediator of viral susceptibility and viral pathogenesis. dSTORM allowed them to detect the interaction between viral P3 protein and microtubules at the level of individual filaments, highlighting dSTORM as a powerful tool for studying virus-host interactions.

## FOCUSED ION BEAM SCANNING ELECTRON MICROSCOPY

The integration of focused ion beam (FIB) technology and scanning electron microscopy (SEM) into a single technique allows researchers to examine subsurface cellular structures at <10 nm resolution (8). Sometimes referred to as “volume electron microscopy,” this 3D imaging technique can provide detailed structural information over large tissue volumes of up to tens of thousands of cubic meters. Once primarily used for materials science and semiconductor research, FIB technology can now be applied to biological samples to mill or dissect biological cells and tissues (38). FIB involves repetitively irradiating samples with gallium (Ga^3+^) ions at intervals of 3–10 nm followed by imaging with SEM to capture sequential images of a biological structure of interest. This generates many images that can be aligned and processed into a 3D rendering of the specimen. Due to the nature of this technique, it is better suited for site-specific investigations of viral and cellular structures that were previously identified using other types of microscopy (i.e., confocal) rather than more open-ended studies.

One intriguing property of many viral infections is their propensity to alter cellular architecture to promote their life cycles. Researchers have turned to focused ion beam scanning electron microscopy (FIB-SEM) to visualize these cellular rearrangements in the context of infection. For example, infection by the simian polyomavirus, SV40, reorganizes the endoplasmic reticulum (ER) membrane into focus structures that are essential for productive infection (39). These virus-induced structures are the site of ER membrane penetration into the cytosol, a key step that is necessary for this DNA virus to reach the host cell cytosol, where it interacts with other cellular factors for trafficking to the nucleus (40, 41). In one report, Bagchi et al. used FIB-SEM to investigate the molecular architecture of this site and determine why it is ideally suited for the site of ER exit (42). Analysis of sliced images taken of the foci identified a flower-like structure that is composed of multi-tubular ER junctions with SV40 localized to the center of the structure. From this, the authors identified that lunapark, an ER membrane protein that has a role in stabilizing ER three-way junctions, is recruited to and stabilizes the foci to assist in viral membrane penetration and cytosolic escape.

Similarly, FIB-SEM has been used to investigate HIV-1 rearrangement of cellular actin during viral assembly and release. Previous literature highlighted the necessity of F-actin remodeling for the spread of HIV by cell-to-cell contacts, but a gap in the field was the mechanism by which HIV induced that remodeling (43, 44). The study by Aggarwal et al. found that FIB-SEM could be used to visualize F-actin structures and membrane curvature, showing the localization of HIV-1 to the tips and ridges of filopodia and lamellipodia (45). These findings suggest that HIV-1 hijacks a pathway related to membrane curvature to facilitate viral spread through cellular contact sites, identifying key host factors that mediate this cytoskeletal rearrangement. Together, these studies illustrate the expert use of FIB-SEM to visualize virus-induced cellular structures and other virus-host interactions that could not have been distinguished using fluorescence microscopy alone.

## CRYOGENIC ELECTRON MICROSCOPY

While traditional electron microscopy has been used to visualize biological materials for several decades, cryogenic electron microscopy has revolutionized the field of structural biology by allowing researchers to achieve near-atomic resolution images (~3 to 4 Å) of their purified samples while maintaining structural integrity (9). Thus, cryogenic electron microscopy (“cryo-EM”) refers to a broad range of experimental techniques that use transmission electron microscopy on plunge-frozen samples to minimize the radiation damage that typically occurs from the interaction of electrons with biological material (46). These advances are possible due to the development of new methods that can rapidly flash-freeze, or vitrify, organic matter in a thin layer of glass-like ice that preserves the sample’s native state.

One of the most commonly used variations of this technique is single-particle cryo-EM, in which data from a large number of 2D projections of an identical protein or complex in different orientations are combined to create an accurate 3D representation of the structure (46). Interestingly, some of the highest-resolution structures ever captured by single-particle cryo-EM have been of icosahedral viruses, such as the adeno-associated virus (1.56 Å) (47), due to their high level of symmetry (60-fold or higher) and relatively large molecular mass (48). This method has been used to solve the structures of numerous viruses that impact human health, including SARS-CoV-2, which caused a global pandemic in 2020 (49). By employing single-particle cryo-EM to solve the structure of the viral spike protein, researchers gained valuable insights into vaccine and therapeutic development (50–52). However, as this review focuses mainly on the use of high-resolution microscopy techniques to identify novel virus-host interactions within the cell, we will focus on a subdiscipline of cryo-EM technology that has been particularly groundbreaking in this area.

## CRYOGENIC ELECTRON TOMOGRAPHY

Cryogenic electron tomography (cryo-ET) is a state-of-the-art method that accurately captures intracellular structures by collecting a series of images taken at different angles relative to the microscope’s electron beam. These images are combined using computational pipelines to generate detailed 3D tomograms of the specimen. Averaging methods can then be applied to further refine the data and provide even more detailed information on the structure of interest (53). Given its ability to capture cellular components in an unstained cell, *in situ* cryo-ET has emerged as a powerful tool for visualizing heterogeneous or “one-of-a-kind” structures within cells. One limitation of this technique is that it can only be used on specimens that are ~0.5 µm or thinner (46). However, researchers can overcome this by combining it with the previously described FIB milling technology under the required cryogenic conditions (54).

Now, cryo-ET is being used to expand the reach of cryo-EM in studying the structures of viruses and virus-like particles, as well as virus-host interactions within an infected cell. Chikungunya virus (CHIKV) is a single-stranded, positive-sense RNA virus that causes severe disease in humans and animals. During entry, CHIKV is endocytosed into early endosomes, where it undergoes conformational changes to the viral E1–E2 heterodimer, which is required for membrane pore formation. This event is necessary for CHIKV nucleocapsid delivery into the cytoplasm and subsequent genome release (55). However, there were limited structural data to describe the sequential conformational changes and membrane remodeling events that occur during this essential step of the viral life cycle. Therefore, researchers turned to cryo-ET to visualize this process and to identify the key conformational changes that occur to support the membrane remodeling events. With this technology, Prasad et al. were able to visualize the intermediate steps of CHIKV membrane fusion, including cell membrane adhesion and viral genome release (56). This study showcases how cryo-ET can be used to gain 3D structural information on dynamic viral processes across time.

Cryo-ET was also employed to solve the mystery of how the IAV is assembled following replication in the nuclei of infected host cells. IAV has a single-stranded, negative-sense RNA genome that is divided into eight segments. Newly synthesized genomic segments are individually encapsidated into distinct ribonucleoprotein complexes (vRNPs) and assembled in a specific 7 + 1 configuration for packaging into new virus particles (57). While previous fluorescent *in situ* hybridization and other super-resolution microscopy studies had shown that vRNPs cluster en route to the plasma membrane, where virus budding occurs (58, 59), the site of vRNP bundle formation and their association with cellular membranous or membrane-less organelles had never before been visualized. Using cryo-ET, Wachsmuth-Melm et al. were able to capture single vRNPs and determine that they are sorted and clustered on Rab11a-containing endomembranes to facilitate downstream virus assembly. This process is assisted by the membrane remodeling capabilities of the viral HA protein, although HA is not explicitly required (60). The author’s use of cryo-ET to track individual vRNPs in relation to the near-native environment of an infected cell has uncovered novel mechanisms of virus assembly that will be important for understanding IAV reassortment and related zoonotic events.

Although powerful, indeed, it can be difficult to distinguish specific molecules and cellular factors using cryo-ET alone. However, new technology that combines the high-resolution capabilities of EM with the molecular specificity of fluorescence labeling has now been developed to overcome this limitation.

## CORRELATIVE LIGHT AND ELECTRON MICROSCOPY

Correlative light and electron microscopy (CLEM) combines the individual strengths of fluorescent light and electron microscopy into a single technique. Specifically, it allows for the multi-color labeling of various proteins, RNAs, and viruses to be viewed within the overall complex organization of the cell. It is performed by either (i) correlating the results from imaging the same sample using separate light and electron microscopes or (ii) performing the imaging on an integrated system that completes both tasks simultaneously (11). In the first method, samples are imaged using fluorescence microscopy prior to EM sample preparation. The use of labeled grids then allows for the subsequent imaging of that region of interest by EM. Even still, matching the observed areas back to each other can be a major challenge and source of imaging bias. Therefore, some researchers have turned to including fiducial markers in their experiments, such as nanoparticles and polymer beads, that can be identified by both light and electron microscopes to aid in this process (61, 62). Integrated CLEM microscopes circumvent this issue by eliminating the need to transfer samples between steps. There are several commercially available systems that offer this technology with either transmission or scanning electron microscopy compatibility (11). Overall, this method has revolutionized biomedical research, including virology, by allowing researchers to now pinpoint a region of interest within a cell and gain high-resolution structural information on that specific area.

Like the previous techniques that we have discussed, CLEM can be used to advance the study of virus-host interactions across various steps of the infectious life cycle, including replication. Most positive-strand RNA viruses replicate their genomes in viral replication factories (63). These are membranous cytoplasmic structures created during viral infection to support efficient and productive viral genome replication (64). Although the 3D architecture of these replication factories has at times been characterized, their molecular composition and temporal development remain largely unknown. CLEM can be a powerful tool for studying this process. For example, hepatitis C virus (HCV) is an RNA virus that creates a membranous web-like structure in infected cells, which is thought to be the site of replication (65). However, the actual composition of these replication sites remained largely unknown as they were thought to be heterogeneous compartments made up of various cellular components. A limitation of studying viral replication steps using traditional immunofluorescence microscopy is the difficulty in definitively describing the subcellular localization of virus-induced interactions. Romero-Brey et al. used a GFP-tagged HCV replicon, allowing them to specifically look at cellular structures that were GFP-positive to specify HCV-positive cells (66). They observed that these cells contained an accumulation of double-membrane vesicles surrounding lipid droplets that were in close proximity to the ER. This supported their overall conclusion that there are complex cellular membrane compartments generated upon HCV infection that are composed of organelle remodeling factors. This work may provide additional insight into the cellular machinery and host pathways that are commonly exploited during RNA viral replication.

Thus far, we have discussed some of the benefits and limitations of the many high-resolution imaging techniques being used to interrogate virus-host interactions in cells. While most examples discuss the use of a single imaging technique, some researchers have combined two or more to better answer their research questions. This is exemplified in the work of Zila et al., who helped solve the mystery of how HIV enters the nucleus where genome integration occurs (67). It has been well established that HIV uses the nuclear pore complex (NPC) to enter the nucleus as a part of its infectious life cycle, but its intact capsid (~60 nm) was considered too large to pass through the diameter of the narrow nuclear pore complex (~39 nm). While it was clear that at least partial uncoating of the capsid is required for genome integration, the extent of uncoating as well as the timing and cellular location of this process were still unclear. Using CLEM and cryo-ET, they showed that intact HIV capsids can penetrate the central channel of the pore and that the NPC scaffold dilates to ~64 nm in diameter, which is large enough to accommodate this viral cargo (67). This paper revealed that in some cell types, namely, human T cells, the diameter of the NPC is much larger than originally reported and that the more constricted diameter from isolated nuclear envelopes may be more relevant to other stress conditions (68).

Researchers are continuing to use these techniques to refine our understanding of HIV nuclear import—a challenging task, given that these events can be spatially scarce, making it difficult to define the complex interplay between HIV and the NPC during nuclear entry. The study conducted by Hou et al. used cryo-FIB milling with integrated cryo-ET and CLEM to more precisely visualize HIV capsids during different stages of nuclear import (69). They identified and characterized four distinct stages that include approaching, docking, traversing, and, finally, importing HIV capsid cores. They also found that the diameter of the pore increases when HIV capsids are inside, suggesting that there are virus-induced changes to the NPC and that both capsid size and elasticity contribute to the efficiency of nuclear import (69). Overall, these two studies showcase that a combination of two or more of these techniques can enhance our understanding of both viral and cellular biology.

## CONCLUDING REMARKS

In this Full Circle review, we highlight the high-resolution imaging techniques that are currently revolutionizing biomedical research and provide examples of how researchers are utilizing these methods to identify and characterize novel virus-host interactions. As imaging methods continue to advance, so will the field of virology, as our ever-evolving ability to visualize what is happening within an infected cell has been, and will remain, key to developing new understanding of these tiny microbes and how they interact with their hosts. Here, we have discussed the benefits and limitations of several techniques, and while some do require advanced machines and imaging cores for data processing, others only require access to more commonly available microscopes. Looking ahead, we expect that more virologists will begin to collaborate in using these technologies to answer their research questions. By combining advanced imaging methods with other molecular biological and biochemical techniques, we can continue to advance our understanding of the underlying mechanisms of virus infection and learn more about the biology of the cells that they infect. Ultimately, this will expand vaccine and therapeutic discovery to improve human health.

## References

[1] Spriggs CC. 2021. mSphere of Influence: viruses-pathogens or expert cell biologists? mSphere 6:e00241-21. doi:10.1128/mSphere.00241-21 33789943 PMC8546703

[2] Procter DJ, Banerjee A, Nukui M, Kruse K, Gaponenko V, Murphy EA, Komarova Y, Walsh D. 2018. The HCMV assembly compartment is a dynamic golgi-derived MTOC that controls nuclear rotation and virus spread. Dev Cell 45:83–100. doi:10.1016/j.devcel.2018.03.010 29634939 PMC5896778

[3] Procter DJ, Furey C, Garza-Gongora AG, Kosak ST, Walsh D. 2020. Cytoplasmic control of intranuclear polarity by human cytomegalovirus. Nature 587:109–114. doi:10.1038/s41586-020-2714-x 32908309 PMC7644666

[4] Wassie AT, Zhao Y, Boyden ES. 2019. Expansion microscopy: principles and uses in biological research. Nat Methods 16:33–41. doi:10.1038/s41592-018-0219-4 30573813 PMC6373868

[5] Demmerle J, Innocent C, North AJ, Ball G, Müller M, Miron E, Matsuda A, Dobbie IM, Markaki Y, Schermelleh L. 2017. Strategic and practical guidelines for successful structured illumination microscopy. Nat Protoc 12:988–1010. doi:10.1038/nprot.2017.019 28406496

[6] Vicidomini G, Bianchini P, Diaspro A. 2018. STED super-resolved microscopy. Nat Methods 15:173–182. doi:10.1038/nmeth.4593 29377014

[7] van de Linde S, Löschberger A, Klein T, Heidbreder M, Wolter S, Heilemann M, Sauer M. 2011. Direct stochastic optical reconstruction microscopy with standard fluorescent probes. Nat Protoc 6:991–1009. doi:10.1038/nprot.2011.336 21720313

[8] Xu CS, Hayworth KJ, Lu Z, Grob P, Hassan AM, García-Cerdán JG, Niyogi KK, Nogales E, Weinberg RJ, Hess HF. 2017. Enhanced FIB-SEM systems for large-volume 3D imaging. eLife 6:e25916. doi:10.7554/eLife.25916 28500755 PMC5476429

[9] Nogales E, Scheres SHW. 2015. Cryo-EM: a unique tool for the visualization of macromolecular complexity. Mol Cell 58:677–689. doi:10.1016/j.molcel.2015.02.019 26000851 PMC4441764

[10] Baumeister W. 2022. Cryo-electron tomography: a long journey to the inner space of cells. Cell 185:2649–2652. doi:10.1016/j.cell.2022.06.034 35868271

[11] de Boer P, Hoogenboom JP, Giepmans BNG. 2015. Correlated light and electron microscopy: ultrastructure lights up! Nat Methods 12:503–513. doi:10.1038/nmeth.3400 26020503

[12] Chen F, Tillberg PW, Boyden ES. 2015. Optical imaging. Expansion microscopy. Science 347:543–548. doi:10.1126/science.1260088 25592419 PMC4312537

[13] Asano SM, Gao R, Wassie AT, Tillberg PW, Chen F, Boyden ES. 2018. Expansion microscopy: protocols for imaging proteins and RNA in cells and tissues. Curr Protoc Cell Biol 80:e56. doi:10.1002/cpcb.56 30070431 PMC6158110

[14] Shepherd RA, Tanaka K, King HAD, Schou MD, Lloyd Williams OH, Kim Y, Roche M, Lewin SR. 2025. Host-directed approaches in the pursuit of a cure for HIV. Antiviral Res 240:106216. doi:10.1016/j.antiviral.2025.106216 40543761 PMC13171291

[15] Petrich A, Hwang GM, La Rocca L, Hassan M, Anders-Össwein M, Sonntag-Buck V, Heuser A-M, Laketa V, Müller B, Kräusslich H-G, Klaus S. 2024. Expanding insights: harnessing expansion microscopy for super-resolution analysis of HIV-1-cell interactions. Viruses 16:1610. doi:10.3390/v16101610 39459943 PMC11512423

[16] Francis AC, Marin M, Singh PK, Achuthan V, Prellberg MJ, Palermino-Rowland K, Lan S, Tedbury PR, Sarafianos SG, Engelman AN, Melikyan GB. 2020. HIV-1 replication complexes accumulate in nuclear speckles and integrate into speckle-associated genomic domains. Nat Commun 11:3505. doi:10.1038/s41467-020-17256-8 32665593 PMC7360574

[17] Müller TG, Zila V, Peters K, Schifferdecker S, Stanic M, Lucic B, Laketa V, Lusic M, Müller B, Kräusslich H-G. 2021. HIV-1 uncoating by release of viral cDNA from capsid-like structures in the nucleus of infected cells. eLife 10:e64776. doi:10.7554/eLife.64776 33904396 PMC8169111

[18] Grover JR, Veatch SL, Ono A. 2015. Basic motifs target PSGL-1, CD43, and CD44 to plasma membrane sites where HIV-1 assembles. J Virol 89:454–467. doi:10.1128/JVI.02178-14 25320329 PMC4301125

[19] de Souza Cardoso R, Murakami T, Jacobovitz B, Veatch SL, Ono A. 2025. PIP_2_ promotes the incorporation of CD43, PSGL-1, and CD44 into nascent HIV-1 particles. Sci Adv 11:eads9711. doi:10.1126/sciadv.ads9711 40184445 PMC11970457

[20] Gustafsson MG, Agard DA, Sedat JW. 1999. I5M: 3D widefield light microscopy with better than 100 nm axial resolution. J Microsc 195:10–16. doi:10.1046/j.1365-2818.1999.00576.x 10444297

[21] Gustafsson MG. 2000. Surpassing the lateral resolution limit by a factor of two using structured illumination microscopy. J Microsc 198:82–87. doi:10.1046/j.1365-2818.2000.00710.x 10810003

[22] Chen X, Zhong S, Hou Y, Cao R, Wang W, Li D, Dai Q, Kim D, Xi P. 2023. Superresolution structured illumination microscopy reconstruction algorithms: a review. Light Sci Appl 12:172. doi:10.1038/s41377-023-01204-4 37433801 PMC10336069

[23] Erickson KD, Bouchet-Marquis C, Heiser K, Szomolanyi-Tsuda E, Mishra R, Lamothe B, Hoenger A, Garcea RL. 2012. Virion assembly factories in the nucleus of polyomavirus-infected cells. PLoS Pathog 8:e1002630. doi:10.1371/journal.ppat.1002630 22496654 PMC3320610

[24] Heiser K, Nicholas C, Garcea RL. 2016. Activation of DNA damage repair pathways by murine polyomavirus. Virology (Auckl) 497:346–356. doi:10.1016/j.virol.2016.07.028 PMC5026627 27529739

[25] Peters DK, Garcea RL. 2020. Murine polyomavirus DNA transitions through spatially distinct nuclear replication subdomains during infection. PLoS Pathog 16:e1008403. doi:10.1371/journal.ppat.1008403 32203554 PMC7117779

[26] Maréchal A, Zou L. 2015. RPA-coated single-stranded DNA as a platform for post-translational modifications in the DNA damage response. Cell Res 25:9–23. doi:10.1038/cr.2014.147 25403473 PMC4650586

[27] Lukinavičius G, Alvelid J, Gerasimaitė R, Rodilla-Ramirez C, Nguyễn VT, Vicidomini G, Bottanelli F, Han KY, Testa I. 2024. Stimulated emission depletion microscopy. Nat Rev Methods Primers 4:56. doi:10.1038/s43586-024-00335-1

[28] Revelo NH, Rizzoli SO. 2015. Application of STED microscopy to cell biology questions. Methods Mol Biol 1251:213–230. doi:10.1007/978-1-4939-2080-8_12 25391802

[29] Baharom F, Thomas OS, Lepzien R, Mellman I, Chalouni C, Smed-Sörensen A. 2017. Visualization of early influenza A virus trafficking in human dendritic cells using STED microscopy. PLoS One 12:e0177920. doi:10.1371/journal.pone.0177920 28591131 PMC5462357

[30] Edinger TO, Pohl MO, Stertz S. 2014. Entry of influenza A virus: host factors and antiviral targets. J Gen Virol 95:263–277. doi:10.1099/vir.0.059477-0 24225499

[31] Bhardwaj N, Bender A, Gonzalez N, Bui LK, Garrett MC, Steinman RM. 1994. Influenza virus-infected dendritic cells stimulate strong proliferative and cytolytic responses from human CD8+ T cells. J Clin Invest 94:797–807. doi:10.1172/JCI117399 8040335 PMC296160

[32] Brisse ME, Ly H. 2019. Hemorrhagic fever-causing arenaviruses: lethal pathogens and potent immune suppressors. Front Immunol 10:372. doi:10.3389/fimmu.2019.00372 30918506 PMC6424867

[33] Byford O, Shaw AB, Tse HN, Moon-Walker A, Saphire EO, Whelan SPJ, Stacey M, Hewson R, Fontana J, Barr JN. 2024. Lymphocytic choriomeningitis arenavirus utilises intercellular connections for cell to cell spread. Sci Rep 14:28961. doi:10.1038/s41598-024-79397-w 39578605 PMC11584850

[34] Heilemann M, van de Linde S, Schüttpelz M, Kasper R, Seefeldt B, Mukherjee A, Tinnefeld P, Sauer M. 2008. Subdiffraction-resolution fluorescence imaging with conventional fluorescent probes. Angew Chem Int Ed Engl 47:6172–6176. doi:10.1002/anie.200802376 18646237

[35] Endesfelder U, Heilemann M. 2015. Direct stochastic optical reconstruction microscopy (dSTORM). Methods Mol Biol 1251:263–276. doi:10.1007/978-1-4939-2080-8_14 25391804

[36] Moseley GW, Lahaye X, Roth DM, Oksayan S, Filmer RP, Rowe CL, Blondel D, Jans DA. 2009. Dual modes of rabies P-protein association with microtubules: a novel strategy to suppress the antiviral response. J Cell Sci 122:3652–3662. doi:10.1242/jcs.045542 19773364

[37] Brice A, Whelan DR, Ito N, Shimizu K, Wiltzer-Bach L, Lo CY, Blondel D, Jans DA, Bell TDM, Moseley GW. 2016. Quantitative analysis of the microtubule interaction of rabies virus P3 protein: roles in immune evasion and pathogenesis. Sci Rep 6:33493. doi:10.1038/srep33493 27649849 PMC5030706

[38] Drobne D. 2013. 3D imaging of cells and tissues by focused ion beam/scanning electron microscopy (FIB/SEM). Methods Mol Biol 950:275–292. doi:10.1007/978-1-62703-137-0_16 23086881

[39] Dupzyk A, Tsai B. 2016. How polyomaviruses exploit the ERAD machinery to cause infection. Viruses 8:242. doi:10.3390/v8090242 27589785 PMC5035956

[40] Ravindran MS, Bagchi P, Inoue T, Tsai B. 2015. A non-enveloped virus hijacks host disaggregation machinery to translocate across the endoplasmic reticulum membrane. PLoS Pathog 11:e1005086. doi:10.1371/journal.ppat.1005086 26244546 PMC4526233

[41] Tsai B. 2007. Penetration of nonenveloped viruses into the cytoplasm. Annu Rev Cell Dev Biol 23:23–43. doi:10.1146/annurev.cellbio.23.090506.123454 17456018

[42] Bagchi P, Liu X, Cho WJ, Tsai B. 2021. Lunapark-dependent formation of a virus-induced ER exit site contains multi-tubular ER junctions that promote viral ER-to-cytosol escape. Cell Rep 37:110077. doi:10.1016/j.celrep.2021.110077 34879280 PMC8759150

[43] Jolly C, Kashefi K, Hollinshead M, Sattentau QJ. 2004. HIV-1 cell to cell transfer across an Env-induced, actin-dependent synapse. J Exp Med 199:283–293. doi:10.1084/jem.20030648 14734528 PMC2211771

[44] Gladnikoff M, Shimoni E, Gov NS, Rousso I. 2009. Retroviral assembly and budding occur through an actin-driven mechanism. Biophys J 97:2419–2428. doi:10.1016/j.bpj.2009.08.016 19883584 PMC2770610

[45] Aggarwal A, Stella AO, Henry CC, Narayan K, Turville SG. 2022. Embedding of HIV egress within cortical F-actin. Pathogens 11:56. doi:10.3390/pathogens11010056 35056004 PMC8777837

[46] Milne JLS, Borgnia MJ, Bartesaghi A, Tran EEH, Earl LA, Schauder DM, Lengyel J, Pierson J, Patwardhan A, Subramaniam S. 2013. Cryo-electron microscopy--a primer for the non-microscopist. FEBS J 280:28–45. doi:10.1111/febs.12078 23181775 PMC3537914

[47] Xie Q, Yoshioka CK, Chapman MS. 2020. Adeno-associated virus (AAV-DJ)-cryo-EM structure at 1.56 Å resolution. Viruses 12:1194. doi:10.3390/v12101194 33092282 PMC7589773

[48] Grigorieff N, Harrison SC. 2011. Near-atomic resolution reconstructions of icosahedral viruses from electron cryo-microscopy. Curr Opin Struct Biol 21:265–273. doi:10.1016/j.sbi.2011.01.008 21333526 PMC3088881

[49] Ke Z, Oton J, Qu K, Cortese M, Zila V, McKeane L, Nakane T, Zivanov J, Neufeldt CJ, Cerikan B, Lu JM, Peukes J, Xiong X, Kräusslich H-G, Scheres SHW, Bartenschlager R, Briggs JAG. 2020. Structures and distributions of SARS-CoV-2 spike proteins on intact virions. Nature 588:498–502. doi:10.1038/s41586-020-2665-2 32805734 PMC7116492

[50] Shang J, Ye G, Shi K, Wan Y, Luo C, Aihara H, Geng Q, Auerbach A, Li F. 2020. Structural basis of receptor recognition by SARS-CoV-2. Nature 581:221–224. doi:10.1038/s41586-020-2179-y 32225175 PMC7328981

[51] Walls AC, Park Y-J, Tortorici MA, Wall A, McGuire AT, Veesler D. 2020. Structure, function, and antigenicity of the SARS-CoV-2 spike glycoprotein. Cell 181:281–292. doi:10.1016/j.cell.2020.02.058 32155444 PMC7102599

[52] Barnes CO, Jette CA, Abernathy ME, Dam K-M, Esswein SR, Gristick HB, Malyutin AG, Sharaf NG, Huey-Tubman KE, Lee YE, Robbiani DF, Nussenzweig MC, West AP Jr, Bjorkman PJ. 2020. SARS-CoV-2 neutralizing antibody structures inform therapeutic strategies. Nature 588:682–687. doi:10.1038/s41586-020-2852-1 33045718 PMC8092461

[53] Navarro PP. 2022. Quantitative cryo-electron tomography. Front Mol Biosci 9:934465. doi:10.3389/fmolb.2022.934465 35874617 PMC9296768

[54] Rigort A, Bäuerlein FJB, Villa E, Eibauer M, Laugks T, Baumeister W, Plitzko JM. 2012. Focused ion beam micromachining of eukaryotic cells for cryoelectron tomography. Proc Natl Acad Sci USA 109:4449–4454. doi:10.1073/pnas.1201333109 22392984 PMC3311327

[55] Lanzrein M, Schlegel A, Kempf C. 1994. Entry and uncoating of enveloped viruses. Biochem J 302 (Pt 2):313–320. doi:10.1042/bj3020313 8092981 PMC1137229

[56] Mangala Prasad V, Blijleven JS, Smit JM, Lee KK. 2022. Visualization of conformational changes and membrane remodeling leading to genome delivery by viral class-II fusion machinery. Nat Commun 13:4772. doi:10.1038/s41467-022-32431-9 35970990 PMC9378758

[57] Noda T, Sagara H, Yen A, Takada A, Kida H, Cheng RH, Kawaoka Y. 2006. Architecture of ribonucleoprotein complexes in influenza A virus particles. Nature 439:490–492. doi:10.1038/nature04378 16437116

[58] Lakdawala SS, Wu Y, Wawrzusin P, Kabat J, Broadbent AJ, Lamirande EW, Fodor E, Altan-Bonnet N, Shroff H, Subbarao K. 2014. Influenza a virus assembly intermediates fuse in the cytoplasm. PLoS Pathog 10:e1003971. doi:10.1371/journal.ppat.1003971 24603687 PMC3946384

[59] Touizer E, Sieben C, Henriques R, Marsh M, Laine RF. 2021. Application of super-resolution and advanced quantitative microscopy to the spatio-temporal analysis of influenza virus replication. Viruses 13:233. doi:10.3390/v13020233 33540739 PMC7912985

[60] Wachsmuth-Melm M, Peterl S, O’Riain A, Makroczyová J, Fischer K, Krischuns T, Vale-Costa S, Amorim MJ, Chlanda P. 2025. Visualizing influenza A virus assembly by in situ cryo-electron tomography. Nat Commun 16:9394. doi:10.1038/s41467-025-65117-z 41130956 PMC12550032

[61] Sochacki KA, Shtengel G, van Engelenburg SB, Hess HF, Taraska JW. 2014. Correlative super-resolution fluorescence and metal-replica transmission electron microscopy. Nat Methods 11:305–308. doi:10.1038/nmeth.2816 24464288 PMC3943662

[62] Kukulski W, Schorb M, Welsch S, Picco A, Kaksonen M, Briggs JAG. 2012. Precise, correlated fluorescence microscopy and electron tomography of lowicryl sections using fluorescent fiducial markers. Methods Cell Biol 111:235–257. doi:10.1016/B978-0-12-416026-2.00013-3 22857932

[63] Shulla A, Randall G. 2016. (+) RNA virus replication compartments: a safe home for (most) viral replication. Curr Opin Microbiol 32:82–88. doi:10.1016/j.mib.2016.05.003 27253151 PMC4983521

[64] Miller S, Krijnse-Locker J. 2008. Modification of intracellular membrane structures for virus replication. Nat Rev Microbiol 6:363–374. doi:10.1038/nrmicro1890 18414501 PMC7096853

[65] Egger D, Wölk B, Gosert R, Bianchi L, Blum HE, Moradpour D, Bienz K. 2002. Expression of hepatitis C virus proteins induces distinct membrane alterations including a candidate viral replication complex. J Virol 76:5974–5984. doi:10.1128/jvi.76.12.5974-5984.2002 12021330 PMC136238

[66] Romero-Brey I, Merz A, Chiramel A, Lee J-Y, Chlanda P, Haselman U, Santarella-Mellwig R, Habermann A, Hoppe S, Kallis S, Walther P, Antony C, Krijnse-Locker J, Bartenschlager R. 2012. Three-dimensional architecture and biogenesis of membrane structures associated with hepatitis C virus replication. PLoS Pathog 8:e1003056. doi:10.1371/journal.ppat.1003056 23236278 PMC3516559

[67] Zila V, Margiotta E, Turoňová B, Müller TG, Zimmerli CE, Mattei S, Allegretti M, Börner K, Rada J, Müller B, Lusic M, Kräusslich H-G, Beck M. 2021. Cone-shaped HIV-1 capsids are transported through intact nuclear pores. Cell 184:1032–1046. doi:10.1016/j.cell.2021.01.025 33571428 PMC7895898

[68] Zimmerli CE, Allegretti M, Rantos V, Goetz SK, Obarska-Kosinska A, Zagoriy I, Halavatyi A, Hummer G, Mahamid J, Kosinski J, Beck M. 2021. Nuclear pores dilate and constrict in cellulo. Science 374:eabd9776. doi:10.1126/science.abd9776 34762489

[69] Hou Z, Fronik S, Shen Y, Chen L, Thompson C, Neumann S, Zhang P. 2025. Direct visualization of HIV-1 core nuclear import and its interplay with the nuclear pore. EMBO Rep 26:5133–5153. doi:10.1038/s44319-025-00567-6 40883508 PMC12592377

